# Supplementary material for: Association between oral microbiome and seven types of cancers in East Asian population: a two-sample Mendelian randomization analysis
Source: Front Mol Biosci. 2023 Nov 21;10:1327893. doi: 10.3389/fmolb.2023.1327893 (PMC10702768; doi:10.3389/fmolb.2023.1327893)
Supplement: Supplementary file 1 [file DataSheet1.ZIP › Supplementary Materials/MR plots of saliva/saliva═╝/Prostate cancer/pheno.861_to_prostate cancer_forest.pdf]

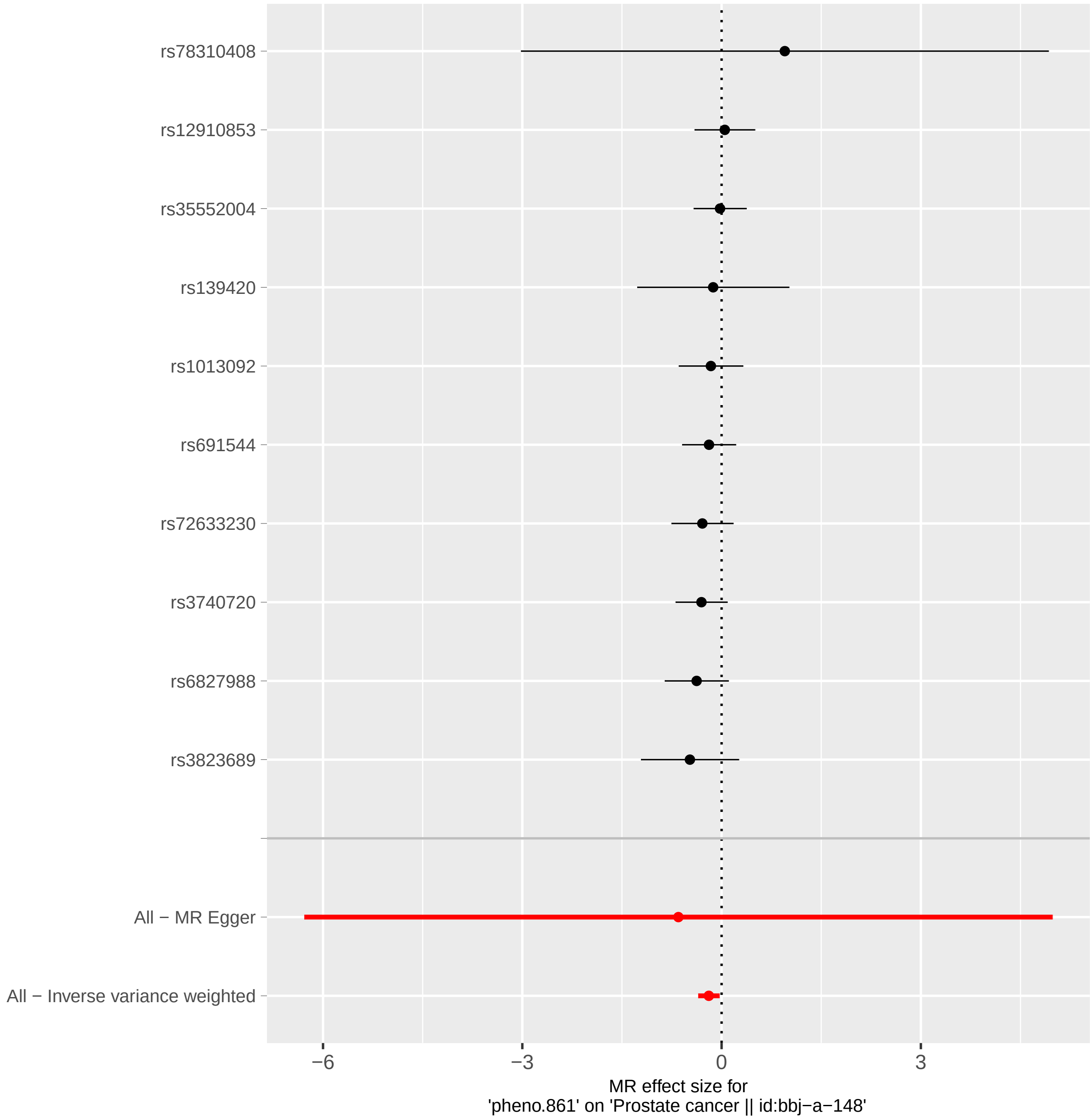
rs78310408
rs12910853
rs35552004
rs139420
rs1013092
rs691544
rs72633230
rs3740720
rs6827988
rs3823689
All − MR Egger
All − Inverse variance weighted
−6
−3
0
3
MR effect size for
'pheno.861' on 'Prostate cancer || id:bbj−a−148'
